# *N*′-[(*E*)-4-Hydr­oxy-3-methoxy­benzyl­idene]pyridine-4-carbohydrazide

**DOI:** 10.1107/S1600536809044134

**Published:** 2009-10-28

**Authors:** Zahid Shafiq, Muhammad Yaqub, M. Nawaz Tahir, Abid Hussain, M. Saeed Iqbal

**Affiliations:** a Department of Chemistry, Bahauddin Zakariya University, Multan-60800, Pakistan; b Department of Chemistry, Bahauddin Zakariya University, Multan 60800, Pakistan; c Department of Physics, University of Sargodha, Sargodha, Pakistan; d Department of Chemistry, Government College University, Lahore, Pakistan

## Abstract

In the title compound, C_14_H_13_N_3_O_3_, the two six-membered rings are oriented at a dihedral angle of 15.17 (11)° and an intra­molecular O—H⋯O hydrogen bond occurs. In the crystal, mol­ecules inter­act by way of N—H⋯O, O—H⋯N and C—H⋯O hydrogen bonds, thereby generating *S*(5) chain and *R*_2_^1^(7) ring motifs.

## Related literature

For related structures, see: Liu & Shi (2007 ▶); Shi *et al.* (2007 ▶); Shafiq *et al.* (2009 ▶). For graph-set theory, see: Bernstein *et al.* (1995 ▶).
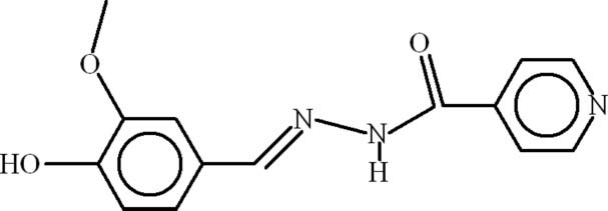


## Experimental

### 

#### Crystal data

C_14_H_13_N_3_O_3_
*M* *_r_* = 271.27
Monoclinic, 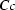

*a* = 14.8543 (10) Å
*b* = 12.4943 (9) Å
*c* = 7.7162 (5) Å
β = 116.716 (2)°
*V* = 1279.20 (15) Å^3^
*Z* = 4
Mo *K*α radiation
μ = 0.10 mm^−1^
*T* = 296 K
0.32 × 0.14 × 0.10 mm

#### Data collection

Bruker Kappa APEXII CCD diffractometer
Absorption correction: multi-scan (*SADABS*; Bruker, 2005 ▶) *T*_min_ = 0.973, *T*_max_ = 0.984
7060 measured reflections
1613 independent reflections
1431 reflections with *I* > 2σ(*I*)
*R*_int_ = 0.028

#### Refinement

*R*[*F* ^2^ > 2σ(*F* ^2^)] = 0.034
*wR*(*F* ^2^) = 0.086
*S* = 1.04
1613 reflections
183 parameters
2 restraints
H-atom parameters constrained
Δρ_max_ = 0.16 e Å^−3^
Δρ_min_ = −0.21 e Å^−3^

### 

Data collection: *APEX2* (Bruker, 2007 ▶); cell refinement: *SAINT* (Bruker, 2007 ▶); data reduction: *SAINT*; program(s) used to solve structure: *SHELXS97* (Sheldrick, 2008 ▶); program(s) used to refine structure: *SHELXL97* (Sheldrick, 2008 ▶); molecular graphics: *ORTEP-3 for Windows* (Farrugia, 1997 ▶) and *PLATON* (Spek, 2009 ▶); software used to prepare material for publication: *WinGX* (Farrugia, 1999 ▶) and *PLATON*.

## Supplementary Material

Crystal structure: contains datablocks global, I. DOI: 10.1107/S1600536809044134/hb5177sup1.cif

Structure factors: contains datablocks I. DOI: 10.1107/S1600536809044134/hb5177Isup2.hkl

Additional supplementary materials:  crystallographic information; 3D view; checkCIF report

## Figures and Tables

**Table 1:** Hydrogen-bond geometry (Å, °)

*D*—H⋯*A*	*D*—H	H⋯*A*	*D*⋯*A*	*D*—H⋯*A*
O2—H2*B*⋯O3	0.82	2.25	2.694 (2)	114
N2—H2*A*⋯O1^i^	0.86	2.25	3.089 (2)	164
O2—H2*B*⋯N1^ii^	0.82	1.96	2.703 (3)	150
C5—H5⋯O1^i^	0.93	2.55	3.410 (3)	153

Symmetry codes: (i) 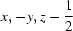; (ii) 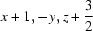.

**Fig. 1.**
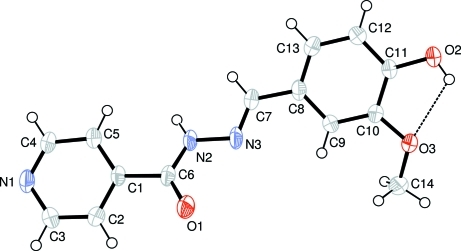
View of (I) with displacement ellipsoids drawn at the 50% probability level. H-atoms are shown by spheres of arbitrary radius. The dotted line represent the intramolecular H-bondings.

**Fig. 2.**
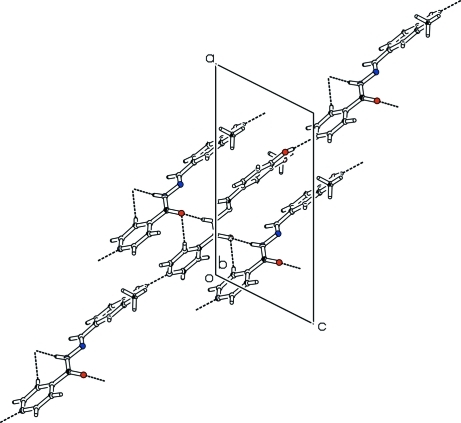
The partial packing of (I), which shows that molecules form two dimensional polymeric chains.

## supplementary crystallographic information

### Comment

We have reported the crystal structures of (II) *N*'-[(*E*)-(4-Hydroxy-3-methoxyphenyl)methylidene]benzohydrazide (Shafiq *et al.*, 2009). The title compound (I, Fig. 1), has been prepared in continuation of synthesizing hydrazide derivatives.

The crystal structure of (III) *N*'-(4-Hydroxy-3-methoxybenzylidene)isonicotinohydrazide monohydrate (Shi *et al.*, 2007) and (IV) *N*'-(4-Hydroxy-3-methoxybenzylidene)isonicotinohydrazide methanol solvate (Liu & Shi, 2007) have also been reported. The title compound differs from (III) and (IV) as there is no solvate.

In the title compound the pyridine ring A (C1–C3/N1/C4/C5) and the benzene ring of vanilline B (C8—C13) are planar with a maximum r. m. s. deviations of 0.0061 and 0.0122 Å respectively, from their mean square planes. The dihedral angle between A/B is 15.17 (11)°. The intramolecular H-bonding of O—H···O type completes S(5) ring motif (Bernstein *et al.*, 1995). There also exist *R*_2_^1^(7) ring motif due to intermolecular H-bondings of C—H···O and N—H···O type (Table 1, Fig. 2). The molecules are stabilized in the form of two dimensional polymeric sheets owing to intermolecular H-bondings of O—H···N type (Fig. 2).

### Experimental

To a hot stirred solution of isoniazid (1.37 g, 0.01 mol) in ethanol (15 ml) was added vanillin (1.52 g, 0.01 mol). The resultant mixture was then heated under reflux. After an hour precipitates were formed. The reaction mixture was further heated about 30 min for the completion of the reaction which was monitored through TLC. The reaction mixture was cooled to room temperature, filtered and washed with hot ethanol. Yellow needles of (I) were obtained by recrystallization of the crude product in 1,4-dioxan:ethanol (1:1) after two days.

### Refinement

In the absence of significant anomalous dispersion effects, Friedel pairs were averaged before refinement.

The H-atoms were positioned geometrically (O–H = 0.82 Å, N–H = 0.86 Å, C–H = 0.93–0.96 Å) and refined as riding with *U*_iso_(H) = 1.2*U*_eq_(carrier) or 1.5*U*_eq_(methyl C).

### Crystal data

**Table:**

C_14_H_13_N_3_O_3_	*F*(000) = 568
*M**_r_* = 271.27	*D*_x_ = 1.409 Mg m^−^^3^
Monoclinic, *C**c*	Mo *K*α radiation, λ = 0.71073 Å
Hall symbol: C -2yc	Cell parameters from 1613 reflections
*a* = 14.8543 (10) Å	θ = 2.2–28.7°
*b* = 12.4943 (9) Å	µ = 0.10 mm^−^^1^
*c* = 7.7162 (5) Å	*T* = 296 K
β = 116.716 (2)°	Needle, yellow
*V* = 1279.20 (15) Å^3^	0.32 × 0.14 × 0.10 mm
*Z* = 4	

### Data collection

**Table:**

Bruker Kappa APEXII CCD diffractometer	1613 independent reflections
Radiation source: fine-focus sealed tube	1431 reflections with *I* > 2σ(*I*)
graphite	*R*_int_ = 0.028
Detector resolution: 7.40 pixels mm^-1^	θ_max_ = 28.7°, θ_min_ = 2.2°
ω scans	*h* = −17→19
Absorption correction: multi-scan (*SADABS*; Bruker, 2005)	*k* = −16→16
*T*_min_ = 0.973, *T*_max_ = 0.984	*l* = −10→5
7060 measured reflections	

### Refinement

**Table:**

Refinement on *F*^2^	Primary atom site location: structure-invariant direct methods
Least-squares matrix: full	Secondary atom site location: difference Fourier map
*R*[*F*^2^ > 2σ(*F*^2^)] = 0.034	Hydrogen site location: inferred from neighbouring sites
*wR*(*F*^2^) = 0.086	H-atom parameters constrained
*S* = 1.04	*w* = 1/[σ^2^(*F*_o_^2^) + (0.0491*P*)^2^ + 0.2451*P*] where *P* = (*F*_o_^2^ + 2*F*_c_^2^)/3
1613 reflections	(Δ/σ)_max_ < 0.001
183 parameters	Δρ_max_ = 0.16 e Å^−^^3^
2 restraints	Δρ_min_ = −0.21 e Å^−^^3^

### Special details

**Table:**

Geometry. Bond distances, angles *etc*. have been calculated using the rounded fractional coordinates. All su's are estimated from the variances of the (full) variance-covariance matrix. The cell e.s.d.'s are taken into account in the estimation of distances, angles and torsion angles
Refinement. Refinement of *F*^2^ against ALL reflections. The weighted *R*-factor *wR* and goodness of fit *S* are based on *F*^2^, conventional *R*-factors *R* are based on *F*, with *F* set to zero for negative *F*^2^. The threshold expression of *F*^2^ > σ(*F*^2^) is used only for calculating *R*-factors(gt) *etc*. and is not relevant to the choice of reflections for refinement. *R*-factors based on *F*^2^ are statistically about twice as large as those based on *F*, and *R*- factors based on ALL data will be even larger.

### Fractional atomic coordinates and isotropic or equivalent isotropic displacement parameters (Å^2^)

**Table:**

	*x*	*y*	*z*	*U*_iso_*/*U*_eq_	
O1	0.20978 (12)	−0.14131 (15)	0.1499 (2)	0.0464 (5)	
O2	0.74733 (14)	0.23723 (14)	0.7114 (3)	0.0495 (5)	
O3	0.71022 (13)	0.02563 (13)	0.6995 (3)	0.0462 (5)	
N1	−0.10456 (14)	−0.14377 (17)	−0.4734 (3)	0.0415 (6)	
N2	0.24963 (13)	−0.01999 (16)	−0.0200 (2)	0.0357 (5)	
N3	0.34327 (13)	−0.00082 (17)	0.1376 (3)	0.0371 (5)	
C1	0.08684 (14)	−0.10723 (18)	−0.1727 (3)	0.0302 (6)	
C2	0.03464 (17)	−0.2001 (2)	−0.1794 (3)	0.0400 (7)	
C3	−0.06004 (17)	−0.2154 (2)	−0.3320 (4)	0.0455 (8)	
C4	−0.05475 (16)	−0.0540 (2)	−0.4634 (3)	0.0387 (6)	
C5	0.04083 (16)	−0.03215 (18)	−0.3174 (3)	0.0342 (6)	
C6	0.18841 (15)	−0.09215 (18)	−0.0012 (3)	0.0317 (6)	
C7	0.38334 (15)	0.08629 (19)	0.1254 (3)	0.0355 (6)	
C8	0.48092 (15)	0.12099 (18)	0.2766 (3)	0.0322 (6)	
C9	0.54708 (16)	0.05029 (18)	0.4162 (3)	0.0338 (6)	
C10	0.63784 (15)	0.08723 (18)	0.5602 (3)	0.0320 (6)	
C11	0.66190 (15)	0.19614 (18)	0.5701 (3)	0.0318 (6)	
C12	0.59690 (16)	0.26504 (19)	0.4295 (3)	0.0353 (6)	
C13	0.50728 (15)	0.22751 (19)	0.2826 (3)	0.0360 (6)	
C14	0.70271 (18)	−0.08742 (19)	0.6725 (4)	0.0424 (7)	
H2	0.06282	−0.25144	−0.08249	0.0480*	
H2A	0.23175	0.01414	−0.12720	0.0428*	
H2B	0.77967	0.18945	0.78640	0.0594*	
H3	−0.09420	−0.27850	−0.33636	0.0546*	
H4	−0.08569	−0.00289	−0.55979	0.0465*	
H5	0.07325	0.03162	−0.31695	0.0411*	
H7	0.34970	0.12947	0.01696	0.0425*	
H9	0.53013	−0.02163	0.41231	0.0405*	
H12	0.61341	0.33710	0.43342	0.0424*	
H13	0.46457	0.27431	0.18744	0.0432*	
H14A	0.70409	−0.10498	0.55254	0.0636*	
H14B	0.64062	−0.11205	0.66859	0.0636*	
H14C	0.75836	−0.12140	0.77800	0.0636*	

### Atomic displacement parameters (Å^2^)

**Table:**

	*U*^11^	*U*^22^	*U*^33^	*U*^12^	*U*^13^	*U*^23^
O1	0.0372 (9)	0.0570 (11)	0.0298 (8)	−0.0070 (8)	0.0015 (7)	0.0075 (7)
O2	0.0341 (8)	0.0373 (9)	0.0468 (9)	−0.0080 (7)	−0.0086 (7)	0.0022 (8)
O3	0.0347 (8)	0.0346 (9)	0.0433 (9)	−0.0031 (7)	−0.0056 (7)	0.0066 (7)
N1	0.0238 (8)	0.0486 (12)	0.0369 (10)	−0.0013 (8)	0.0001 (7)	−0.0017 (8)
N2	0.0252 (9)	0.0448 (11)	0.0234 (8)	−0.0053 (8)	−0.0013 (7)	0.0011 (7)
N3	0.0233 (8)	0.0481 (11)	0.0253 (8)	−0.0042 (8)	−0.0021 (7)	−0.0012 (8)
C1	0.0226 (9)	0.0371 (11)	0.0235 (9)	−0.0011 (8)	0.0037 (8)	−0.0041 (8)
C2	0.0317 (12)	0.0392 (12)	0.0366 (11)	−0.0011 (9)	0.0042 (10)	0.0068 (10)
C3	0.0302 (12)	0.0441 (14)	0.0484 (13)	−0.0087 (10)	0.0054 (10)	0.0025 (11)
C4	0.0258 (10)	0.0455 (13)	0.0316 (10)	0.0017 (9)	0.0011 (8)	0.0037 (9)
C5	0.0260 (10)	0.0387 (12)	0.0295 (10)	−0.0028 (9)	0.0050 (8)	−0.0021 (9)
C6	0.0242 (9)	0.0376 (12)	0.0240 (9)	0.0021 (8)	0.0027 (8)	−0.0028 (8)
C7	0.0257 (10)	0.0421 (12)	0.0281 (10)	−0.0004 (9)	0.0028 (8)	0.0005 (9)
C8	0.0230 (9)	0.0403 (12)	0.0269 (9)	−0.0028 (9)	0.0055 (8)	−0.0015 (9)
C9	0.0280 (10)	0.0311 (11)	0.0327 (10)	−0.0059 (8)	0.0052 (8)	0.0006 (8)
C10	0.0258 (9)	0.0326 (11)	0.0291 (9)	−0.0010 (8)	0.0048 (8)	0.0027 (8)
C11	0.0242 (9)	0.0343 (11)	0.0296 (10)	−0.0037 (8)	0.0055 (8)	−0.0018 (8)
C12	0.0323 (11)	0.0304 (11)	0.0358 (11)	−0.0033 (9)	0.0087 (9)	−0.0007 (9)
C13	0.0295 (11)	0.0387 (12)	0.0305 (10)	0.0035 (9)	0.0053 (9)	0.0039 (9)
C14	0.0352 (12)	0.0342 (12)	0.0485 (13)	0.0013 (10)	0.0105 (10)	0.0065 (10)

### Geometric parameters (Å, °)

**Table:**

O1—C6	1.227 (3)	C8—C13	1.382 (3)
O2—C11	1.349 (3)	C8—C9	1.398 (3)
O3—C10	1.366 (3)	C9—C10	1.385 (3)
O3—C14	1.425 (3)	C10—C11	1.400 (3)
O2—H2B	0.8200	C11—C12	1.382 (3)
N1—C3	1.334 (3)	C12—C13	1.386 (3)
N1—C4	1.327 (3)	C2—H2	0.9300
N2—C6	1.334 (3)	C3—H3	0.9300
N2—N3	1.396 (3)	C4—H4	0.9300
N3—C7	1.264 (3)	C5—H5	0.9300
N2—H2A	0.8600	C7—H7	0.9300
C1—C5	1.381 (3)	C9—H9	0.9300
C1—C2	1.384 (3)	C12—H12	0.9300
C1—C6	1.506 (3)	C13—H13	0.9300
C2—C3	1.383 (4)	C14—H14A	0.9600
C4—C5	1.386 (3)	C14—H14B	0.9600
C7—C8	1.459 (3)	C14—H14C	0.9600
			
C10—O3—C14	117.5 (2)	O2—C11—C10	122.6 (2)
C11—O2—H2B	109.00	C10—C11—C12	119.5 (2)
C3—N1—C4	117.5 (2)	C11—C12—C13	120.5 (2)
N3—N2—C6	118.82 (17)	C8—C13—C12	120.5 (2)
N2—N3—C7	113.6 (2)	C1—C2—H2	120.00
C6—N2—H2A	121.00	C3—C2—H2	120.00
N3—N2—H2A	121.00	N1—C3—H3	119.00
C2—C1—C5	118.3 (2)	C2—C3—H3	119.00
C2—C1—C6	117.45 (19)	N1—C4—H4	118.00
C5—C1—C6	124.2 (2)	C5—C4—H4	118.00
C1—C2—C3	119.1 (2)	C1—C5—H5	121.00
N1—C3—C2	123.0 (2)	C4—C5—H5	121.00
N1—C4—C5	123.7 (2)	N3—C7—H7	119.00
C1—C5—C4	118.5 (2)	C8—C7—H7	119.00
O1—C6—N2	123.0 (2)	C8—C9—H9	120.00
N2—C6—C1	116.85 (18)	C10—C9—H9	120.00
O1—C6—C1	120.1 (2)	C11—C12—H12	120.00
N3—C7—C8	121.9 (2)	C13—C12—H12	120.00
C7—C8—C13	118.5 (2)	C8—C13—H13	120.00
C7—C8—C9	122.0 (2)	C12—C13—H13	120.00
C9—C8—C13	119.5 (2)	O3—C14—H14A	109.00
C8—C9—C10	120.1 (2)	O3—C14—H14B	109.00
O3—C10—C9	125.6 (2)	O3—C14—H14C	109.00
O3—C10—C11	114.4 (2)	H14A—C14—H14B	109.00
C9—C10—C11	120.0 (2)	H14A—C14—H14C	109.00
O2—C11—C12	118.0 (2)	H14B—C14—H14C	110.00
			
C14—O3—C10—C9	14.0 (4)	N1—C4—C5—C1	−0.7 (4)
C14—O3—C10—C11	−165.5 (2)	N3—C7—C8—C9	16.8 (4)
C4—N1—C3—C2	−0.6 (4)	N3—C7—C8—C13	−162.1 (2)
C3—N1—C4—C5	1.3 (4)	C7—C8—C9—C10	−178.3 (2)
C6—N2—N3—C7	−162.0 (2)	C13—C8—C9—C10	0.6 (4)
N3—N2—C6—O1	1.4 (3)	C7—C8—C13—C12	176.7 (2)
N3—N2—C6—C1	177.92 (19)	C9—C8—C13—C12	−2.3 (4)
N2—N3—C7—C8	179.5 (2)	C8—C9—C10—O3	−177.3 (2)
C5—C1—C2—C3	1.5 (4)	C8—C9—C10—C11	2.2 (4)
C6—C1—C2—C3	178.3 (2)	O3—C10—C11—O2	−3.1 (3)
C2—C1—C5—C4	−0.8 (3)	O3—C10—C11—C12	176.2 (2)
C6—C1—C5—C4	−177.4 (2)	C9—C10—C11—O2	177.4 (2)
C2—C1—C6—O1	−21.5 (3)	C9—C10—C11—C12	−3.4 (4)
C2—C1—C6—N2	161.9 (2)	O2—C11—C12—C13	−179.0 (2)
C5—C1—C6—O1	155.1 (2)	C10—C11—C12—C13	1.7 (4)
C5—C1—C6—N2	−21.5 (3)	C11—C12—C13—C8	1.1 (4)
C1—C2—C3—N1	−0.8 (4)		

### Hydrogen-bond geometry (Å, °)

**Table:**

*D*—H···*A*	*D*—H	H···*A*	*D*···*A*	*D*—H···*A*
O2—H2B···O3	0.82	2.25	2.694 (2)	114
N2—H2A···O1^i^	0.86	2.25	3.089 (2)	164
O2—H2B···N1^ii^	0.82	1.96	2.703 (3)	150
C5—H5···O1^i^	0.93	2.55	3.410 (3)	153

Symmetry codes: (i) *x*, −*y*, *z*−1/2; (ii) *x*+1, −*y*, *z*+3/2.
